# Health and economic consequences of different options for timing the coordinated global cessation of the three oral poliovirus vaccine serotypes

**DOI:** 10.1186/s12879-015-1113-7

**Published:** 2015-09-17

**Authors:** Kimberly M. Thompson, Radboud J. Duintjer Tebbens

**Affiliations:** Kid Risk, Inc, 10524 Moss Park Rd., Ste. 204-364, Orlando, FL 32832 USA

## Abstract

**Background:**

World leaders remain committed to globally-coordinated oral poliovirus vaccine (OPV) cessation following successful eradication of wild polioviruses, but the best timing and strategy for implementation depend on existing and emerging conditions.

**Methods:**

Using an existing integrated global poliovirus risk management model, we explore alternatives to the current timing plan of coordinated cessation of each OPV serotype (i.e., OPV1, OPV2, and OPV3 cessation for serotypes 1, 2, and 3, respectively). We assume the current timing plan involves OPV2 cessation in 2016 followed by OPV1 and OPV3 cessation in 2019 and we compare this to alternative timing options, including cessation of all three serotypes in 2018 or 2019, and cessation of both OPV2 and OPV3 in 2017 followed by OPV1 in 2019.

**Results:**

If Supplemtal Immunization Activity frequency remains sufficiently high through cessation of the last OPV serotype, then all OPV cessation timing options prevent circulating vaccine-derived poliovirus (cVDPV) outbreaks after OPV cessation of any serotype. The various OPV cessation timing options result in relatively modest differences in expected vaccine-associated paralytic poliomyelitis cases and expected total of approximately 10–13 billion polio vaccine doses used. However, the expected amounts of vaccine of different OPV formulations needed changes dramatically with each OPV cessation timing option. Overall health economic impacts remain limited for timing options that only change the OPV formulation but preserve the currently planned year for cessation of the last OPV serotype and the global introduction of inactivated poliovirus vaccine (IPV) introduction. Earlier cessation of the last OPV serotype or later global IPV introduction yield approximately $1 billion in incremental net benefits due to saved vaccination costs, although the logistics of implementation of OPV cessation remain uncertain and challenging.

**Conclusions:**

All countries should maintain the highest possible levels of population immunity to transmission for each poliovirus serotype prior to the coordinated cessation of the OPV serotype to manage cVDPV risks. If OPV2 cessation gets delayed, then global health leaders should consider other OPV cessation timing options.

## Background

The Global Polio Eradication Initiative (GPEI) continues to make progress toward the primary goal of interrupting all global transmission of the three wild poliovirus (WPV) serotypes [1]. After apparent interruption of indigenous serotype 2 WPV (WPV2) by 2000 [2] and no serotype 3WPV (WPV3) detections since late 2012 [3], the GPEI primarily focuses on interrupting serotype 1 WPV (WPV1) transmission. Only 3 countries reported poliomyelitis cases due to indigenous WPV1 s in 2014 (i.e., Afghanistan, Nigeria, and Pakistan) but polio-free countries with insufficient population immunity remain at risk of outbreaks due to imported WPV1 as long as circulation continues anywhere [1]. In almost all countries, WPV elimination resulted from aggressive use of oral poliovirus vaccine (OPV) in both routine immunization (RI) and supplemental immunization activities (SIAs), which provides humoral and intestinal immunity to recipients and their close contacts. However, the live, attenuated OPV vaccine comes with a risk of vaccine-associated paralytic poliomyelitis (VAPP) disease in a very small fraction of vaccine recipients and close contacts [4]. Moreover, populations with very low immunity may allow OPV viruses to continue to circulate and eventually evolve to become circulating vaccine-derived polioviruses (cVDPVs) with properties similar to WPVs. Multiple cVDPV outbreaks (defined as transmission that led to 1 or more cases of paralytic poliomyelitis) occurred to date [5–8], including re-established (cVDPV2) in Northern Nigeria since 2005 [9, 10]. In addition, long-term infection and potential excretion from immunodeficiency-associated VDPVs (iVDPVs), which occur in a small number of poliovirus-infected patients with B-cell-related primary immunodeficiency disease can pose a risk of reintroduction of polioviruses [6, 7, 11–13]. Ending the use of OPV stops the creation of new cVDPVs, iVDPVs, and VAPP cases, and OPV cessation represents the only means to eliminate the risks and poliomyelitis cases associated with OPV use [7, 14].

OPV comes in different formulations. Trivalent OPV (tOPV) protects against all three serotypes and represents the current vaccine of choice for OPV RI and the vaccine used for SIAs up until 2004. In 2005, the GPEI began using serotype 1 monovalent OPV (mOPV1) and later some serotype 3 (mOPV3) for some SIAs, because of their higher seroconversion (i.e., take rates) for the mOPV serotype than achieved by the same serotype component of tOPV, particularly for the first dose [15, 16]. In 2010, the GPEI turned to using bivalent OPV (bOPV, serotypes 1 and 3), which provides higher take rates for both serotypes 1 and 3 than achieved by the serotype 1 and 3 components of tOPV [15, 16]. However, the reduced reliance on tOPV for SIAs created immunity gaps for serotype 2, which allowed numerous cVDPV2 emergences in different countries [8]. The GPEI currently responds to cVDPV2 outbreaks with tOPV, which takes at a similar rate for serotype 2 as mOPV2 [15, 16]. Given the strong evidence of WPV2 eradication and the ongoing burden of paralytic poliomyelitis caused by cVDPV2s, the GPEI plans to first globally-coordinate stopping the use of serotype 2-containing OPV (i.e., OPV2 cessation), followed by cessation of all OPV containing the two other serotypes (i.e., OPV13 cessation) after global WPV eradication of all three serotypes [17]. Synchronizing global OPV cessation of any serotype remains critical because countries that still use OPV pose a risk of reintroducing live poliovirus into countries that already stopped using OPV [18, 19].

The inactivated poliovirus vaccine (IPV) currently represents the only vaccine alternative to OPV. While the killed, injectable IPV provides very good individual protection from paralytic poliomyelitis and boosts intestinal immunity in individuals with immunity from a prior live poliovirus infection (LPV, i.e., WPV, VDPV, OPV, or OPV-related virus), it does not protect as well against asymptomatic participation in poliovirus transmission as OPV, particularly in settings with more intense fecal-oral transmission, and it does not spread beyond the vaccine recipient [20–28]. Consequently, in populations with predominantly fecal-oral poliovirus transmission, serotype-specific population immunity to transmission will drop significantly after OPV cessation of that serotype, regardless of IPV use [29, 30]. The drop in population immunity after OPV cessation implies the need to achieve the highest possible population immunity to transmission at the time of OPV cessation to avoid cVDPV outbreaks after OPV cessation [29, 30]. In places with intense fecal-oral transmission and/or poor RI coverage, prior to stopping vaccination with OPV for one or more serotypes, countries should achieve high enough population immunity to transmission by conducting pre-cessation SIAs using OPV containing the serotype(s) to be stopped [29].

Recognizing the risks associated with OPV2 cessation, the GPEI established 6 prerequisites for OPV2 cessation, including the need to validate the elimination of persistent cVDPV2s [17], which requires high population immunity to transmission for serotype 2 [31]. The reality of recent or ongoing cVDPV2 transmission in countries already facing significant challenges with completing and verifying WPV eradication (i.e., Nigeria and Pakistan) [31, 32] as well as long lead times associated with ensuring sufficient tOPV supply for use in SIAs before OPV2 cessation, raise questions about the feasibility of OPV2 cessation by the target date of April 1, 2016 [33]. However, at this point in time, delaying planned OPV2 cessation would come with some reputation and programmatic risks. Moreover, the apparent but uncertain global eradication of WPV3 [3] and increasing confidence about its true disappearance as time increases since the last observed case [31], open up the possibility of OPV3 cessation at the same time as OPV2 cessation (i.e., OPV23 cessation). Alternatively, if apparent WPV1 elimination occurs before the world meets the prerequisites for OPV2 cessation, then cessation of all 3 serotypes of OPV (i.e., OPV123 cessation) may offer a means to combine the substantial logistics of phased OPV cessation (i.e., OPV2 followed by OPV13 cessation) into a single event (i.e., OPV123 cessation), which would also potentially simplify vaccine choices up until OPV123 cessation. Despite the importance of the choices, no prior studies have characterized the health or economic impacts of different OPV cessation timing options. This study uses modeling to explore the health and economic implications of different OPV cessation timing options and the associated numbers of different formulations of poliovirus vaccines needed for each option.

## Methods

We use an integrated global model of long-term poliovirus risk management options (i.e., the global model) described in detail elsewhere [34]. Briefly, the model relies on a deterministic, dynamic poliovirus transmission and OPV evolution model [35, 36] to track immunity, poliovirus incidence, and any cVDPV emergences in populations. We determined model input values based on an extensive expert review and model calibration process [22, 26, 35–37]. The expert review process elicited ranges of numerical values for generic (i.e., not setting-specific) model inputs to characterize immunity states, waning, and OPV evolution based on the expert interpretation of the evidence [8, 22, 37]. The model calibration process ensured internal consistency and found model inputs within the elicited ranges that produced behavior consistent with the evidence of numerous aspects of poliovirus transmission (i.e., paralytic incidence during endemic transmission and outbreaks, WPV elimination, recipient and contact VAPP incidence, secondary OPV spread and evolution, cVDPV emergence in some settings but not in others, age distributions of cases, serology, and serotype differences) across 10 different situations (i.e., countries or parts of countries) [26, 35, 36]. To estimate recipient VAPP cases, the poliovirus transmission and OPV evolution model tracks OPV infections (i.e., OPV vaccinations that take) in susceptible people (i.e., fully susceptible individuals and a fraction of infants born with maternal immunity) and multiplies these with serotype-specific paralysis-to-infection ratios for OPV [35]. The model assumes that the paralysis-to-infection ratios depend on the serotype but not the OPV formulation, such that differences in VAPP incidence for tOPV, bOPV, and mOPV depend on vaccine used, the difference in serotype-specific take rates, and the resulting probability of acquiring immunity from OPV receipt versus infection from exposure to an LPV [35]. To estimate contact VAPP cases, the model tracks all infections in fully susceptible people with OPV-related viruses that did not yet evolve to fully-reverted VDPVs, and assumes that the serotype-specific VAPP rates increase logarithmically with each of 19 stages of reversion toward the serotype-specific paralysis-to-infection rates for VDPV (assumed to be equivalent to homotypic WPV) [35]. Thus, contact VAPP cases include all paralytic infections from OPV and OPV-related viruses, but not from fully-reverted VDPV, which the model classifies as cVDPV or iVDPV outbreak cases, depending on the source of the virus.

The global model [34] estimates the number of polio cases that occur and the costs of different long-term poliovirus risk management options compared with the continued *status quo* of OPV use in most countries to characterize incremental cost-effectiveness ratios (ICERs) and incremental net benefits (INBs) using 2013 United States dollars ($). The global model estimates financial costs associated with RI and SIAs, including any outbreak response SIAs (oSIAs) before and after OPV cessation [34]. The global model assumes that surveillance, treatment, and other programmatic activities remain the same for the main policy options [34] and the alternative OPV cessation timing options considered in this analysis. We adopt all of the economic inputs from the global model [34], including a 3 % discount rate, treatment and societal economic costs per paralytic case, disability-adjusted life-years (DALYs) averted per prevented paralytic case, and vaccination cost inputs. We model the costs for different OPV vaccine formulations as equivalent [34] and emphasize that IPV costs significantly more than OPV, with some differences by income level. We report ICERS and INBs from a societal perspective in 2015 for each alternative OPV cessation timing option compared to the current timing plan of OPV2 cessation in April 2016 followed by OPV13 cessation in April 2019, based on expected costs and cases during 2015–2019.

To model the global population and poliovirus transmission, the global model [34] assumes 710 subpopulations of approximately 10 million people (in 2013) that mix homogenously in space and heterogeneously by age. The global model further groups these 710 subpopulations into 71 blocks consisting of 10 subpopulations and randomly generates exportations of poliovirus from subpopulations, assuming the majority remain within the block. We base the epidemiological, demographic, and transmission assumptions for the blocks and subpopulations on the conditions that exist in the world in 2013 [34]. The global model further generates randomly occurring poliovirus reintroduction events from different sources after OPV cessation [7], including iVDPVs [13] and releases of seed strains from IPV production sites. However, given our focus for this analysis on the more predictable short-term outcomes (i.e., VAPP cases and cVDPV emergences), we ignore these stochastic risks here.

Table 1 lists the different OPV cessation timing options modeled. We assume that all OPV cessation timing options involve a global minimum recommendation of at least 1 added IPV RI dose for at least 5 years after cessation of the last OPV serotype in all blocks that used only OPV for RI in 2013 (i.e., 52 of 71 blocks). Unless otherwise noted, the model assumes that blocks that use only OPV in 2013 add 1 IPV RI dose co-administered with OPV at the scheduled age of the third non-birth OPV RI dose (low- or lower middle-income blocks) or switch to a sequential IPV/OPV schedule (upper middle-income blocks) on January 1, 2015. At the time of cessation of the last OPV serotype, all blocks that already switched to a sequential IPV/OPV schedule move to an IPV-only RI schedule, while blocks that only include a single IPV dose added to a primary OPV RI schedule continue with a single IPV RI dose [34]. The global model adopts previously developed methods to characterize the different RI schedules [35, 38]. Specifically, in the context of uncertainty about the age at which partially covered children (i.e., those that do not receive the full non-birth RI schedule, but receive at least one non-birth dose) receive their polio dose(s), the schedule of 1 added IPV RI dose co-administered with OPV assumes that all children who receive at least one non-birth OPV RI dose also receive the IPV dose [38].

**Table 1:** OPV cessation timing options considered

Name of OPV cessation timing option	OPV1 cessation	OPV2 cessation	OPV3 cessation	Addition of IPV dose(s)^a^	RI vaccine^b^	SIA vaccine(s)^c^
Current timing plan	April 1, 2019	April 1, 2016	April 1, 2019	January 1, 2015	tOPV before OPV2 cessation, then bOPV until OPV13 cessation	tOPV and bOPV before OPV2 cessation, then only bOPV until OPV13 cessation
OPV23 cessation in 2017	April 1, 2019	April 1, 2017	April 1, 2017	January 1, 2015	tOPV before OPV23 cessation, then mOPV1 until OPV1 cessation	tOPV and bOPV before OPV23 cessation, then only mOPV1 until OPV1 cessation
OPV123 cessation in 2018	April 1, 2018	April 1, 2018	April 1, 2018	January 1, 2015	tOPV until all-OPV cessation	tOPV and bOPV until OPV123 cessation
OPV123 cessation in 2019	April 1, 2019	April 1, 2019	April 1, 2019	January 1, 2015	tOPV until all-OPV cessation	tOPV and bOPV until OPV123 cessation
OPV123 cessation in 2019 with tOPV-only from 2017	April 1, 2019	April 1, 2019	April 1, 2019	January 1, 2015	tOPV until all-OPV cessation	tOPV and bOPV until April 1, 2017, then only tOPV until OPV123 cessation
OPV123 cessation in 2019 with IPV added from 2018	April 1, 2019	April 1, 2019	April 1, 2019	January 1, 2018	tOPV until all-OPV cessation	tOPV and bOPV until OPV123 cessation

*Abbreviations*: bOPV, bivalent OPV (serotypes 1 and 3); IPV, inactivated poliovirus vaccine; mOPV1, serotype 1 monovalent OPV; OPV, oral poliovirus vaccine; OPV(##) cessation, globally-coordinated cessation of OPV containing the serotype(s) indicated by ##; RI, routine immunization; SIA, supplemental immunization activity; tOPV, trivalent OPV

^a^Assumes IPV co-administered with third non-birth OPV RI dose from time of addition of IPV dose until cessation of the last OPV serotype, followed by a single-IPV-dose schedule for low- and lower middle-income blocks; assumes a sequential IPV/IPV/OPV/OPV schedule from time of addition of IPV doses until cessation of the last OPV serotypes followed by IPV/IPV/IPV schedule in upper middle-income blocks that use OPV-only in 2013; assumes a sequential IPV/IPV/OPV/OPV schedule from the beginning of the analytical time horizon until cessation of the last OPV serotypes followed by an IPV/IPV/IPV schedule in upper middle- and high-income blocks that already use IPV/OPV in 2013; assumes an IPV/IPV/IPV schedule for all years in high-income countries that already use IPV-only in 2013 [34]

^b^In blocks that use OPV or IPV/OPV for RI in 2013

^c^In blocks that use OPV or IPV/OPV in 2013 and require SIAs, not including any mOPV used for outbreak response SIAs that override any planned, preventive SIAs [34]

All options assume tOPV intensification from January 1, 2015 characterized by an increased proportion of planned, preventive SIAs (pSIAs, which excludes all oSIAs) using tOPV instead of bOPV [34, 39]. The current timing plan (i.e., *status quo*) option assumes OPV2 cessation on April 1, 2016, consistent with the current target date [33], and OPV13 cessation on April 1, 2019, consistent with the anticipated window [17]. For the current timing plan option, we assume that RI and all tOPV pSIAs switch to bOPV after OPV2 cessation until OPV13 cessation. We also consider the option of combining OPV2 and OPV3 cessation, which continues the use of all OPV serotypes (i.e., tOPV) until April 1, 2017, and then switches all RI and pSIA vaccine to mOPV1 on April 1, 2017 (OPV23 cessation in 2017). For simultaneous cessation of all three OPV serotypes, we consider options stopping on April 1, 2018 (i.e., OPV123 cessation in 2018) or 2019 (i.e., OPV123 cessation in 2019), for which we assume continued tOPV use for RI and continuation of the pSIA vaccine mix of tOPV and bOPV associated with tOPV intensification [34, 39] until OPV123 cessation. We explore a variation that starts the same way but assumes tOPV use exclusively from April 1, 2017 until April 1, 2019 (i.e., OPV123 cessation in 2019 with tOPV-only from 2017). The deterministic run-up of the global model leads to global eradication of all WPVs in early 2016, and thus the latter scenario assumes that approximately 1 year after global WPV interruption the focus would shift equally to all 3 serotypes by using only tOPV. Finally, we consider a variation of OPV123 cessation in 2019 with delayed global IPV introduction on January 1, 2018 (i.e., OPV123 cessation in 2019 with IPV added from 2018) instead of the current timing plan assumption of global IPV introduction on January 1, 2015, although countries increasingly continue to include IPV in their RI schedules. To estimate numbers of doses of vaccine used, we adopt effective wastage estimates from the global model [34, 39], which for SIAs include both actual wastage and a demographic uncertainty factor of 1.5 to account for differences in the United Nations World Population Prospects estimates [40] and the estimates used by the GPEI in SIA planning.

## Results

Due to the assumed sufficiently high frequency of SIAs leading up to OPV cessation of any serotype for all OPV cessation options, none of the options lead to any subsequent cVDPV cases. With only a small number of paralytic cases from WPV1 remaining in the model during the first year of the time horizon (i.e., 4 in 2015), VAPP cases represent the main source of paralytic poliomyelitis in the model for 2015–2019. Table 2 lists the expected total number of VAPP cases for each of the OPV cessation timing options, along with the number of tOPV, bOPV, mOPV1, and IPV vaccine doses used during this time period. With respect to the VAPP cases, the duration of OPV use of each serotype and IPV use represent the main drivers. For example, the current timing plan results in the fewest serotype 2 VAPP cases because it stops all serotype 2-containing vaccine use one year earlier than the other options considered. OPV23 cessation in 2017 yields the fewest VAPP cases overall, because in 2017 it removes the two serotypes most frequently associated with VAPP [35]. While co-administration of IPV with OPV in low and lower middle-income blocks results in a negligible impact on VAPP, switching to a sequential IPV/OPV schedule in the 19 upper middle-income blocks that used OPV-only in 2013 results in a large reduction, notable in the difference in global VAPP totals between OPV123 cessation in 2019 with IPV added in 2018 and OPV123 cessation in 2019 (i.e., with IPV added in 2015 as planned).

**Table 2:** Estimated (undiscounted) VAPP cases and OPV vaccine use by serotype for the different OPV cessation timing options that all lead to no expected cVDPV cases

Name of OPV cessation timing option	Number of VAPP cases 2015-2019				Polio vaccine use 2015–2019 (billions of doses)^a^				
	PV1	PV2	PV3	Total	tOPV	bOPV	mOPV1	IPV	All polio vaccine
Current timing plan	41	75	404	520	3.3	8.2	0	1.3	12.8
OPV23 cessation in 2017	42	122	219	383	5.2	1.3	4.9	1.3	12.8
OPV123 cessation in 2018	34	168	302	504	7.1	1.9	0	1.4	10.4
OPV123 cessation in 2019	43	214	383	634	9.0	2.5	0	1.3	12.8
OPV123 cessation in 2019 with tOPV-only from 2017	44	210	382	635	10.1	1.3	0	1.3	12.8
OPV123 cessation in 2019 with IPV added from 2018	58	252	499	810	9.2	2.5	0	0.8	12.4

*Abbreviations*: bOPV, bivalent OPV (serotypes 1 and 3); cVPDV, circulating vaccine-derived poliovirus; IPV, inactivated poliovirus vaccine; mOPV1, serotype 1 monovalent OPV; OPV, oral poliovirus vaccine; OPV(##) cessation, globally-coordinated cessation of OPV containing the serotype(s) indicated by ##; PV(1,2,3), poliovirus (serotype 1, 2, or 3, respectively); RI, routine immunization; SIA, supplemental immunization activity; tOPV, trivalent OPV; VAPP, vaccine-associated paralytic poliomyelitis

^a^Includes RI and planned, preventive SIAs, with no outbreak response SIAs triggered for any of the options

With respect to vaccine dose estimates, our results include the entire world (i.e., not just countries supported by the GPEI), assume relatively high effective wastage, and assume sustained high SIA frequency up until cessation of the last OPV serotype [39]. Most scenarios result in the same number of 12.8 billion total polio vaccine doses over the 5-year time period, including 11.4 billion OPV doses (89 %) and approximately 1.3 billion IPV doses (11 %). However, OPV123 cessation in 2018 implies one fewer year of OPV use than the other options, which saves 2.5 billion OPV doses. OPV123 cessation in 2019 with IPV added from 2018 involves three years of global IPV use less than the other options, which saves 0.6 billion IPV doses. OPV123 cessation in 2018 involves slightly more IPV doses than all other options with non-delayed global IPV introduction, because earlier OPV cessation of the last serotype implies that blocks with a sequential IPV/OPV schedule move to IPV-only one year earlier than all other options.

Table 3 provides estimates of the ICERs and INBs for the alternative OPV cessation timing options compared to the current timing plan. These estimates include the vaccination costs only, and do not include any programmatic costs for surveillance, laboratories, stockpile, or coordination of OPV cessation, which we assume would generally cancel out in the context of the incremental analyses. All but one of the ICERs involve either costs savings (i.e., negative numerators) or additional incurred paralytic cases (i.e., negative denominators), or both, making the ICER numerically ill-defined [41, 42]. For OPV123 cessation in 2018, in high-income blocks, the incremental costs remain marginally positive due to the earlier move to an all-IPV schedule while preventing less than one VAPP case, leading to a very high ICER of 1.9 million $ per DALY averted due to the very small (i.e., almost 0) denominator.

**Table 3:** Incremental economic outcomes for different OPV cessation timing options compared to the current timing plan based on vaccination costs and expected paralytic polio cases between 2015 and 2019

Alternative	Incremental cost-effectiveness ratio^a^				Incremental net benefits ($)				
	LOW	LMI	UMI	HIGH	LOW	LMI	UMI	HIGH	All
OPV23 cessation in 2017	CLS	CLS	CLS	CLS	0.3	3.0	2.7	1.3	7.3
OPV123 cessation in 2018	CLS	CLS	CLS	1.9	300	660	190	−8.6	1,100
OPV123 cessation in 2019	Dominated	Dominated	Dominated	Dominated	−0.3	−2.6	−1.6	0	−4.6
OPV123 cessation in 2019 with tOPV-only from 2017	Dominated	Dominated	Dominated	Dominated	−0.3	−2.5	−1.6	0	−4.5
OPV123 cessation in 2019 with IPV added from 2018	CSLC	CSLC	CSLC	Dominated	110	370	380	0	860

*Abbreviations*: HIGH, high-income; IPV, inactivated poliovirus vaccine; LMI, lower middle-income; LOW, low-income; OPV, oral poliovirus vaccine; OPV(##) cessation, globally-coordinated cessation of OPV containing the serotype(s) indicated by ##; UMI, upper middle-income; $, 2013 United States dollar

^a^Numbers represent millions of $ per disability-adjusted life-year averted; Letters are: CLS, cost and life-saving (i.e., negative incremental costs and positive cases prevented); CSLC cost-saving but life-costing (i.e., positive incremental costs and negative cases prevented); Dominated, positive incremental costs and negative cases prevented

The INBs represent a more informative metric for this analysis. OPV23 cessation in 2017, OPV123 cessation in 2019, and OPV123 cessation in 2019 with tOPV-only from 2017 all result in the same expected financial costs for vaccination as the current timing plan because they involve different OPV formulations without affecting the overall duration of OPV or IPV use in any of the income levels. Thus, for these alternative OPV cessation timing options the incremental costs reflect only treatment costs (or savings) associated with additional incurred (or prevented) paralytic cases. Given the relatively small number of paralytic cases involved in any of these options, their INBs remain close to 0. The INB estimate of approximately -$4.5 million for OPV123 cessation in 2019 (with or without tOPV-only from 2017) suggests that if the excluded logistical costs of coordinating OPV cessation once instead of twice amount to more than $4 million (and OPV123 cessation in 2018 remains logistically impossible), then some economic justification exists for this original path of coordinated OPV cessation of all three OPV serotypes. OPV123 cessation in 2019 with tOPV-only from 2017 yields almost the same INBs as OPV123 cessation in 2019, which involves some continued bOPV SIAs (Table 2). However, OPV123 cessation with tOPV-only from 2017 results in higher population immunity to transmission for serotype 2 while not significantly reducing population immunity to transmission for serotypes 1 and 3 [43]. Thus, OPV123 cessation with tOPV-only from 2017 offers the potential to further reduce cVDPV2 risks and/or reduce the frequency of SIAs needed to avoid cVDPVs of any serotype after OPV123 cessation [39, 43].

Two OPV cessation timing options result in substantial expected INBs. If logistically possible, OPV123 cessation in 2018 results in INBs of $1.2 billion, because stopping all OPV use one year earlier significantly reduces the total number of vaccinations while also preventing some VAPP cases. However, this option only becomes feasible with very rapid achievement and confidence of interruption of WPV1 transmission. OPV123 cessation in 2019 with IPV added from 2018 results in INBs of approximately $0.9 billion associated with savings from later global IPV introduction. While this option results in the most VAPP cases among the considered options, later global IPV introduction yields cost savings despite the relative low fraction of IPV doses in the total polio vaccine doses used, which occurs due to the comparatively high IPV cost per dose.

## Discussion

Our analysis demonstrates the large number of viable OPV cessation options that avoid cVDPVs after OPV cessation of each serotype, as long as SIA intensity remains high enough. In this context, the overall economic implications of different OPV cessation timing options remain relatively small, unless the options involve a shorter duration of OPV or IPV use globally. Prior experience with polio shows a societal willingness to accept large financial costs to prevent or reduce a relatively small burden of vaccine-associated disease [44], which may favor options that avoid more VAPP cases regardless of small differences in incremental net benefits. The large benefits of potentially stopping all OPV use earlier reinforce the point that earlier global eradication is better, even if it may require more resources in the short term [45]. However, optimally balancing the risk of undetected WPV circulation against the potential benefits of stopping the last OPV serotype earlier remains complex [31]. If delays in cessation of the first OPV serotype (i.e., OPV2) appear likely, then postponing OPV2 cessation until the earliest possible time of OPV13 cessation may offer the advantage of saving the costs associated with coordinating global OPV cessation of at least one serotype twice. However, given that OPV2 viruses evolve more rapidly to become cVDPVs than the other two serotypes [8, 35], longer continuation of OPV2 use may imply a higher frequency of SIAs needed for a longer period of time to avoid cVDPV2 emergence [39]. Thus, the current path of OPV2 cessation before OPV13 cessation may offer some potential to save on SIA costs after OPV2 cessation, although this remains a topic of further research.

Our results related to saving financial costs in the short-term by slightly delaying global introduction of IPV artificially assume perfect coordination of implementation. The significant expected benefits of global IPV introduction for the long-term management of poliovirus risks, including individual protection from potential reintroduction of live polioviruses, support a global commitment to IPV introduction [34]. Nevertheless, the logistics of introducing a new vaccine into the complex array of RI schedules [16] for over 120 countries using OPV-only for RI between 2013 and the date by which all countries introduce IPV remain a challenge. Our analysis of delayed IPV introduction provides a lower bound on cost reduction since many countries already introduced IPV as part of the run up to OPV2 cessation and we do not expect that countries would stop using IPV in the context of a global delay. Aggressive efforts to introduce IPV continue to reveal many issues, and IPV introduction will involve some phase-in due to the logistics of RI system planning and long lead times associated with scale-up of IPV production capacity. Current timing plans that include the introduction of IPV with the third dose of OPV in RI do not promise to provide much protection from cVDPVs or VAPP [30]. In contrast, IPV use prevents VAPP cases if used in a sequential schedule [25, 46], and consequently we suggest that all countries may benefit from considering IPV introduction as an opportunity to change to a sequential IPV/OPV schedule, even if including only a single dose of IPV, to obtain the benefits of VAPP reduction as soon as possible. Further analyses should consider the costs, benefits, and other implications of pursuing this strategy instead of the current strategy of introducing IPV with the third OPV dose.

While our model provides the first estimates of the economic implications of different OPV cessation timing options, several limitations may impact our results. First, we assume continued high SIA frequency up until cessation of the last OPV serotype, which includes assumptions that may prove optimistic for the timing of global WPV eradication and number of WPV cases. More WPV cases in reality than currently modeled would not necessarily affect incremental economic metrics (i.e., ICERs and INBs), because the cases would occur for all OPV cessation timing options. However, longer transmission of WPV1 and/or cVDPV2 will affect the feasibility of some of the options and expected SIA frequency and costs. Some reduction in SIA frequency may remain sufficient to keep population immunity high enough to avoid cVDPVs after OPV cessation, although too much reduction or inconsistency in SIA quality would result in cVDPVs [39], and insufficient immunization in Pakistan, particularly in the under-vaccinated (and inaccessible) subpopulation, will lead to a delay in WPV1 eradication and/or meeting the current pre-requisites for OPV2 cessation. Second, significant uncertainty about effective vaccine wastage led us to include a demographic uncertainty factor of 1.5, which significantly increases dose estimates and costs [39]. Better estimates of demographics and vaccine tracking would lead to improved estimates. Third, we did not explicitly account for programmatic costs not related to vaccination, including the costs to coordinate OPV cessation, which represents a large logistical challenge. However, we assumed that these costs would apply to all of the options we modeled and thus cancel out in the incremental economic metrics. The small difference in INBs between options with one or two OPV cessation events suggests that combining cessation of all OPV serotypes may offer some cost savings that this analysis does not capture. Fourth, the global situation continues to evolve, and this leads to some discrepancies between the model and the most up-to-date expected current path. For example, China, which represents half of the population in all upper middle-income countries, will likely not adopt an IPV/IPV/OPV/OPV sequential schedule in 2015 but transition to this schedule by first adopting an IPV/OPV/OPV/OPV schedule, which results in lower costs. Similarly, many countries may not introduce IPV by the end of 2015 due to the logistical challenges and time delays associated with global IPV supply phase-in and production. Fifth, the global model assumes that all children in low and lower middle-income blocks who receive at least one non-birth OPV RI dose do so at the age of the third non-birth RI OPV dose so that they also receive a co-administered IPV dose [34, 38]. This assumption may somewhat overestimate IPV-induced immunity and IPV costs if in reality only children who receive 3 or more OPV doses receive IPV. Sixth, we did not include the long-term poliovirus risk implications of different OPV cessation timing options. While the impact of different OPV cessation timing options on probabilities of long-term outbreaks likely remains small, even small changes in probabilities can result in large consequences in a fraction of stochastic model realizations [34]. Seventh, we did not address how tightly cessation needs to be coordinated, and further research should address this issue. Finally, all limitations and uncertainties from the global model [34] and the dynamic poliovirus transmission and OPV evolution model [35] also apply to this analysis.

Our analysis leads to the realization that flexibility in national vaccine licensing for the transition period may prove helpful. While under the current plan the GPEI continues to encourage countries to pursue licenses of bOPV for RI use after OPV2 cessation, our analysis suggests potential value in simultaneously pursuing national licenses for RI use of bOPV or mOPV1, and potentially also securing licenses now to use any mOPV serotype for outbreak response if ever needed. In addition, given uncertainty about the status of population immunity for serotype 2, our results underscore the importance of developing contingencies with manufacturers to continue tOPV production past the current planned OPV2 cessation target of April 2016.

## Conclusions

All countries should maintain the highest possible levels of population immunity to transmission for each poliovirus serotype prior to the coordinated cessation of the OPV serotype. If OPV2 cessation gets delayed, then global health leaders should consider other OPV cessation timing options.

## Abbreviations

bOPV: Bivalent oral poliovirus vaccine (serotypes 1 and 3)
CLS: Cost-and life-saving
CSLC: Cost-saving but life-costing
cVDPV(1,2,3): Circulating vaccine-derived poliovirus (serotype 1, 2, or 3, respectively)
DALY: Disability-adjusted life-year
GPEI: Global Polio Eradication Initiative
HIGH: High-income
ICER: Incremental cost-effectiveness ratio
INBs: Incremental net benefits
IPV: Inactivated poliovirus vaccine
iVDPV: Immunodeficiency-associated vaccine-derived poliovirus
LMI: Lower middle-income
LOW: Low-income
LPV: Live poliovirus
mOPV(1,2,3): Monovalent oral poliovirus vaccine (serotype 1, 2, or 3, respectively)
OPV: Oral poliovirus vaccine
OPV(##): cessation Globally-coordinated cessation of OPV containing the serotype(s) indicated by ##
oSIA: Outbreak response SIA
pSIA: Planned, preventive SIA
PV(1,2,3): Poliovirus (serotype 1, 2, or 3, respectively)
RI: Routine immunization
SIA: Supplemental immunization activity
tOPV: Trivalent OPV
UMI: Upper middle-income
VAPP: Vaccine-associated paralytic poliomyelitis
WPV(1,2,3): Wild poliovirus (serotype 1, 2, or 3, respectively)
$: 2013 United States dollars
